# The identification and expression pattern of the sex determination genes and their sex-specific variants in the egg parasitoid *Trichogramma dendrolimi* Matsumura (Hymenoptera: Trichogrammatidae)

**DOI:** 10.3389/fphys.2023.1243753

**Published:** 2023-08-25

**Authors:** Su-Fang Ning, Liang-Xiao Huo, Lin Lv, Ying Wang, Li-Sheng Zhang, Wu-Nan Che, Hui Dong, Jin-Cheng Zhou

**Affiliations:** ^1^ College of Plant Protection, Shenyang Agricultural University, Shenyang, China; ^2^ State Key Laboratory for Biology of Plant Diseases and Insect Pests, Institute of Plant Protection, Chinese Academy of Agricultural Sciences, Beijing, China

**Keywords:** alternative splicing, *dsx*, parasitoid, sex-determining gene, *tra*, *tra2*, *Trichogramma*

## Abstract

**Introduction:** *Trichogramma* wasps are egg parasitoids of agricultural lepidopteran pests. The sex of *Trichogramma* is determined by its ploidy as well as certain sex ratio distorters, such as the endosymbiotic bacteria *Wolbachia* spp. and the paternal sex ratio (PSR) chromosome. The sex determination systems of hymenopterans, such as *Trichogramma* spp., involve cascades of the genes *transformer* (*tra*), *transformer*-*2* (*tra2*), and *doublesex* (*dsx*) and are associated with sex-specific *tra* and *dsx* splicing. First, these genes and their sex-specific variants must be identified to elucidate the interactions between the sex ratio disorders and the sex determination mechanism of *Trichogramma*.

**Methods:** Here, we characterized the sex determination genes *tra*, *tra2*, and *dsx* in *Trichogramma dendrolimi*. Sex-specific *tra* and *dsx* variants were detected in cDNA samples obtained from both male and female *Trichogramma* wasps. They were observed in the early embryos (1–10 h), late embryos (12–20 h), larvae (32 h and 48 h), pre-pupae (96 h), and pupae (144 h, 168 h, 192 h, and 216 h) of both male and female *T. dendrolimi* offspring.

**Results:** We detected female-specific *tra* variants throughout the entire early female offspring stage. The male-specific variant began to express at 9–10 h as the egg was not fertilized. However, we did not find any maternally derived, female-specific *tra* variant in the early male embryo. This observation suggests that the female-specific *tra* variant expressed in the female embryo at 1–9 h may not have originated from the maternal female wasp.

**Discussion:** The present study might be the first to identify the sex determination genes and sex-specific gene splicing in *Trichogramma* wasps. The findings of this study lay the foundation for investigating the sex determination mechanisms of *Trichogramma* and other wasps. They also facilitate sex identification in immature *T. dendrolimi* and the application of this important egg parasitoid in biological insect pest control programs.

## Introduction

*Trichogramma* spp., the egg parasitoids, are efficacious biological control agents against lepidopteran pests in agriculture and forestry (Zhou et al., 2019a; Zang et al., 2020). Female *Trichogramma* wasps oviposit into the eggs of their insect pest hosts (Zhou et al., 2019b). The sex of the *Trichogramma* wasp is determined by its ploidy. Female and male offspring develop from fertilized diploid and unfertilized haploid eggs, respectively (Zhou et al., 2003; Heimpel and de Boer, 2008; Zhang et al., 2020). The sex determination systems of *Trichogramma* are also affected by certain sex ratio distorters, such as the endoparasitic bacteria *Wolbachia* spp. and the paternal sex ratio (PSR) chromosome (Stouthamer et al., 1999; van Vugt et al., 2003; Zhou et al., 2022a). The endosymbiotic bacteria *Wolbachia* spp. induce parthenogenesis in *Trichogramma* wasps by transforming haploid male-destined eggs into diploid embryos during the first mitotic division (Huigens et al., 2000; Zhou et al., 2022b; Zhang et al., 2022). The PSR chromosome causes the generation of male offspring and the loss of the paternal genome during the first mitotic division of the zygote (van Vugt et al., 2003; Dalla Benetta et al., 2020). However, the interactions between the preceding sex ratio disorders and the sex determination systems of *Trichogramma* have not been studied in depth. Therefore, identification and analysis of the sex determination genes in *Trichogramma* wasps will help clarify the interactions between these insects and their sex ratio distorters.

Sex determination systems are usually directed by genetic pathways comprising several sex-determining genes (Wilkins, 1995; Verhulst et al., 2010a; Simoni et al., 2020). Sex determination systems consist of a primary genetic signal, sex-specific signal cascades, and the expression of genes regulating sexual development (Peng et al., 2020). The primary sex-specific signals of hymenopterans are governed by paralog genes designated as transformer (*tra*) or feminizer (*fem*) (Verhulst et al., 2010a). The rapidly evolving *tra* encodes arginine/serine (SR)-rich proteins. Order-specific domains have been detected in the *tra*/*fem* orthologs such as the hymenopteran (HYM) domain in hymenopterans as well as the domains characteristic of other insect orders. The *tra* pre-mRNA can be spliced into female-specific and male-specific variants (Verhulst et al., 2010a; Geuverink et al., 2017a). Only a female-specific *tra* variant produces a functional TRA protein with the Ceritatis-Apis-Musca (CAM) domain and complexes with the protein encoded by *tra2* to regulate female-specific doublesex (*dsx*) splicing (Geuverink et al., 2017b). TRA2 protein is more conserved than TRA. Its RNA-binding domain (RBD) has two regions rich in Ser–Arg (Geuverink et al., 2017b). *Dsx* interprets the sexual traits of various invertebrates (Chikami et al., 2022). The conserved *dsx* contains the DNA-binding motif (DM) domain and is localized to the bottom of the *tra–tra2–dsx* cascade that determines sex in hymenopterans (Verhulst et al., 2010b; Baral et al., 2019).

The haplodiploid sex determination system in hymenopterans is described by the Maternal Effect Genomic Imprinting Sex Determination (MEGISD) model (Beukeboom et al., 2007). In MEGISD, the sex-specific signal involves maternal *tra* mRNA imprinting and the alternative *tra* splicing induced by the zygote (Beukeboom and Kamping, 2006; Verhulst et al., 2010a). In *Nasonia vitripennis*, female-specific *tra* splicing is initiated by timely expression of the paternal wasp *overruler of masculinization* (*wom*) allele upon fertilization (Zou et al., 2020). Sex-specific *tra* cascade *tra2* splicing initiates sexual development by directing *dsx*. In other hymenopterans, offspring sex is determined according to the MEGISD model and is regulated by the complementary sex determination (CSD) mechanism (Matthey-Doret et al., 2019). In CSD, the heterozygotic *csd* locus initiates female development, while the homozygotic *csd* locus promotes diploid male formation (Hagan and Gloag, 2021). CSD proteins direct the sex-specific tra/fem variants, which, in turn, leads to female development (Matthey-Doret et al., 2019; Hagan and Gloag, 2021). Our previous study showed that the CSD mechanism is absent in *Trichogramma dendrolimi* (Liu et al., 2019). The parasitoid wasps *Trichogramma* spp. could be the most important egg parasitoid hosts in biological insect pest control programs (Zang et al., 2020; Zhou et al., 2020). To the best of our knowledge, the sex determination genes and their sex-specific splicing have never been previously identified in *Trichogramma* wasps.

The present study aimed to identify the sex determination genes *tra*, *tra2*, and *dsx*, their sex-specific variants, and their expression patterns at different developmental stages of male and female *Trichogramma dendrolimi* Matsumura. The results of this work could help elucidate the sex determination mechanisms in *Trichogramma* wasps. The output of this study also facilitates sex identification in immature *T. dendrolimi* as it shows how sex-specific *tra* and *dsx* variants may be detected.

## Materials and methods

### Insects

The isofemale *T. dendrolimi* line was established using a single mating pair of wasps. The insects were reared over several generations on the eggs of the rice moth *Corcyra cephalonica* (Lepidoptera: Pyralidae) at 25°C and 70% relative humidity (RH) under a photoperiod of L16:D8. The host eggs were grouped in lots of ∼500, glued onto a white card, and allowed to parasitize the host eggs. The latter were irradiated with ultraviolet (UV) light (TUV 30W UV lamps; Philips, Amsterdam, The Netherlands) for 45 min. These host egg cards were used for parasitization by *T. dendrolimi*.

### Sample collection

A group of 100 adult male or female *T. dendrolimi* wasps was collected. Male *T. dendrolimi* offspring were obtained from host eggs parasitized by virginal female wasps. Mixtures of male and female offspring were obtained from host eggs parasitized by mated female wasps. Immature *T. dendrolimi* cannot be distinguished by sex. They include early embryos (at 1 h, 2 h, 3 h, 4 h, 5 h, 6 h, 7 h, 8 h, 9 h, and 10 h), late embryos (at 12 h, 14 h, 16 h, 18 h, and 20 h), larvae (at 32 h and 48 h), pre-pupae (at 96 h), and pupae (at 144 h, 168 h, 192 h, and 216 h). All the foregoing stages were collected in lots of ∼1,000.

### RNA extraction and cDNA synthesis

The total RNA of each sample was extracted. Samples included embryos, larvae, pupae, and adults. Total RNA of the *T. dendrolimi* wasp was extracted with TRIzol reagent (Invitrogen, Carlsbad, CA, United States). Total RNA (1 µg) was reverse-transcribed using a PrimeScript RT Kit (TaKaRa, Dalian, China) according to the manufacturer’s protocol. The cDNA product was then immediately stored at −80°C until subsequent use.

### Identification and cloning of *tra*, *tra2*, and *dsx* orthologs

**FIGURE 1:**
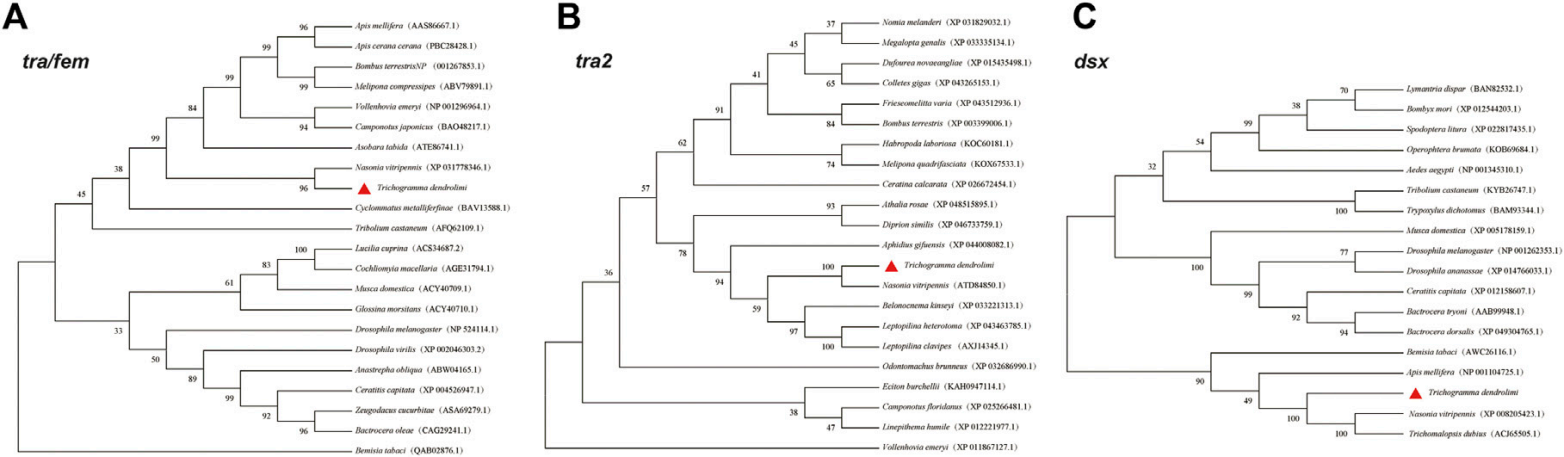
Phylogenetic analysis of amino acid sequences of *tra* **(A)**, *tra2* **(B)**, and *dsx* **(C)** in different insect species.

*tra*, *tra2*, and *dsx* orthologs were identified using the TBLASTN algorithm (https://blast.ncbi.nlm.nih.gov/Blast.cgi?PROGRAM=tblastn&PAGE_TYPE=BlastSearch&LINK_LOC=blasthome) by aligning the *T. dendrolimi* genomic assembly dataset (unpublished data). The study applied the available sequence of TRA/FEM proteins from *N. vitripennis* (NP_001128299), *Apis mellifera* (AAS86667.1), and *Drosophila melanogaster* (NP_524114.1) as the query sequences for the aligning of TRA orthologs. The sequences of TRA2 proteins from *N. vitripennis* (ATD84850.1), *Aphidius gifuensis* (XP_044008082.1), and *Leptopilina clavipes* (AXJ14345.1) were used as the query sequences for identifying TRA2 orthologs. The sequences of DSX proteins from *N. vitripennis* (XP_008205423.1), *A. mellifera* (NP_001104725.1), and *D. melanogaster* (NP_001262353.1) were used as the query sequences for identifying DSX2 orthologs (Figure 1). The start–stop codons and intron–exon boundary of *tra*, *tra2*, and *dsx* were determined using the genome assembly database and the transcriptome constructed by the present group. Similarity analyses were conducted on these genes using Clustal X v. 1.83 (http://www.clustal.org/download/current), Jalview v. 2.10.3 (https://www.jalview.org/download/), and DNAMAN v. 6.0 (https://www.lynnon.com/downloads.html). Primer Premier v. 5.0 (http://www.premierbiosoft.com) was used to design the primers used to amplify the *tra*, *tra2*, and *dsx* transcripts and their sex-specific variants (Table 1). The polymerase chain reaction (PCR) was conducted using 1.0 μL cDNA template and PCR Master Mixture (Promega, Madison, WI, United States) or high-fidelity DNA polymerase (Yeasen, Shanghai, China). The PCR products were confirmed by 1.5% agarose gel electrophoresis, cloned, and sequenced.

**TABLE 1:** Sequences of the primers.

Application of primers	Primer name	Primer sequence (5’→3’)
ORF cloning	*tra*-F	ATG​AGG​CCA​AGT​AAC​AAA​TAC​GA
	*tra*-R	TTA​CGC​GTT​TCT​ATC​ATT​GGC​AT
	*tra2-F*	CGT​GGT​TTA​CTA​CTA​CTC​GT
	*tra2-R*	TAG​GAA​GGT​GAG​AAT​AGC​GA
	*dsx*-F	CGTCGCTAATTGACTTTG
	*dsx*-R	GACTTCGCTTGTGACTTC
RACE cloning	*tra*-5'GSP	GGA​GGA​TTT​GTC​CAT​CGA​CTT​CGA​CTT​G
	*tra*-3'GSP	GAG​TCG​AGT​TTA​CCA​GGT​ATG​AGG​G
	*tra*-UPM-long-primer	CTA​ATA​CGA​CTC​ACT​ATA​GGG​CAA​GCA​GTG​G
		TATCAACGCAGAGT
	*tra*-UPM-short-primer	CTA​ATA​CGA​CTC​ACT​ATA​GGG​C
Alternative splice analysis	*tra*-sex-F	AGA​ACA​TGG​GCC​ACT​CAA​GT
	*tra*-sex-R	TGG​CTT​CTT​TCG​CTG​GAG​TA
	*dsx*-sex-F	TTC​ACA​AGA​GAA​ATT​GCC​CAT​G
	*dsx*-sex-R	ATA​TCT​TCA​GTG​CAG​TTG​AGT

Note: *tra*, *tra2*, and *dsx* indicate the *transformer*, *transformer*-*2*, and *doublesex*, respectively.

### Splicing of sex-specific *tra* and *dsx*

Alternative *tra* and *dsx* variants were amplified using the specific primers listed in Table 1. The PCR products of the alternative splices were confirmed, cloned, and sequenced as previously described. Sex-specific *tra* and *dsx* exons were characterized using the fragments amplified from the cDNA samples of male and female *T. dendrolimi*. The full-length sex-specific transcripts were then merged with the predicted *tra* or *dsx* models for *T. dendrolimi* using the sequenced sex-specific fragments.

### Phylogenetic and molecular evolutionary analyses

Sequences of the homologs in ten insect species from the National Center for Biotechnology Information (NCBI) database (https://www.ncbi.nlm.nih.gov/) were collected to delineate the evolution of *tra*, *tra2*, and *dsx*. A phylogenetic tree was generated using PhyloSuite v. 1.2.2 (Zhang et al., 2020) with the IQ-TREE fitted by the JTT model (Nguyen et al., 2015). Orthologous relationships were identified using 1,000 bootstrap replicates. Conserved domains were identified using the Conserved Domain Database of NCBI.

### Sex-specific splicing expression patterns in *T. dendrolimi*

The primers used for sex-specific *tra* and *dsx* splicing were selected based on the different sequences of female- and male-specific variants and designed using Primer Premier v. 5.0. Thus, the specific regions of female- and male-specific variants can be amplified to different sizes by the single pair of primers. Sex-specific *tra* and *dsx* splicing was validated based on the real-time (RT)-PCR products amplified using the male and female cDNA samples. The dynamic expression of sex-specific *tra* and *dsx* splicing was detected in the male and mixed male–female offspring samples. The RT-PCR products of the sex-specific variants were then detected by 1.5% agarose gel electrophoresis.

## Results

### Identification of *tra*, *tra2*, and *dsx*

**FIGURE 2:**
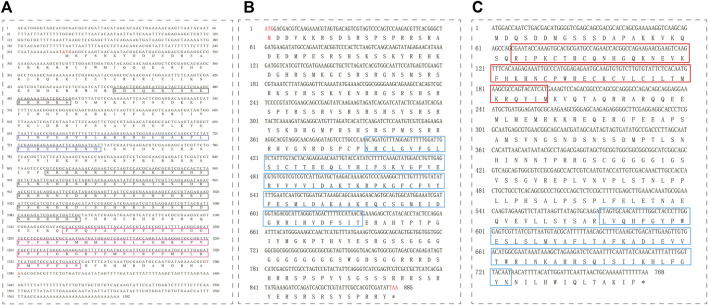
cDNA and amino acid sequences and domains of *tra* **(A)**, *tra2* **(B)**, and *dsx* **(C)** in *T. dendrolimi.* **(A)** The blue box indicates the putative autoregulation domain; the black box indicates the Arg/Ser domain; and the fuchsia box indicates the proline (Pro)-rich region. **(B)** The cyan box indicates the RNA recognition motif (RRM) domain. **(C)** The red box indicates the DNA-binding motif (DM) domain; the cyan box indicates the dimer domain.

The full-length *tra* sequence was 1,592 bp long and included an open reading frame (ORF) of 1,128 bp encoding 375 amino acids (aa) (Table 2). The *tra* aa sequence had the same structure as those of other known insect *tra*. The HYM domain was detected in all *tra*/*fem* orthologs in hymenopterans. All orthologs had a CAM, a proline (Pro)-rich, and an Arg/Ser domain. The phylogenetic tree showed that the aa sequence of *T. dendrolimi tra* was closely related to that of *N. vitripennis tra* (Figure 1; Figure 2; Supplementary Figure S1). Alignment of the aa sequences in *T. dendrolimi* and *N. vitripennis tra* revealed 41.12% identity.

**TABLE 2:** Length of the full sequence, ORF, and protein of *tra*, *tra2*, and *dsx* genes in *T. dendrolimi* and *N. vitripennis*.

Gene	*T. dendrolimi*			*N. vitripennis*			Similarity between proteins
	Full length (bp)	ORF (bp)	Protein (aa)	Full length (bp)	ORF (bp)	Protein (aa)	
*tra*	1,592	1,128	375	1862	1,218	405	41.12%
*tra2*	2,932	855	294	2,690	900	299	76.92%
*dsx*	3,719	768	255	2001	708	235	76.63%

The full-length sequence of *tra2* was 2,932 bp long and included an ORF of 885 bp encoding 294 aa (Table 2). The RBD was detected in the *tra2* orthologs in hymenopterans. The aa sequence of *T. dendrolimi tra2* was similar to that of *N. vitripennis tra2* (Figure 2). Both the *T. dendrolimi* and *N. vitripennis* orthologs had a glycine (Gly)-rich domain (Figure 1; Supplementary Figure S2). Alignment of the aa sequences in *T. dendrolimi* and *N. vitripennis tra2* disclosed 76.92% identity.

The *dsx* ortholog was 3,719 bp long and included an ORF of 768 bp encoding 255 aa (Table 2). The aa sequences of the *T. dendrolimi* and other insect *dsx* orthologs all had the DM and dimer domains (Figure 1; Supplementary Figure S3). The aa sequence of *T. dendrolimi dsx* was similar to that of *N. vitripennis dsx* (Figure 2). Alignment of the aa sequences in *T. dendrolimi* and *N. vitripennis* dsx revealed 76.63% identity.

### Sex-specific variants of *tra* and *dsx*

**FIGURE 3:**
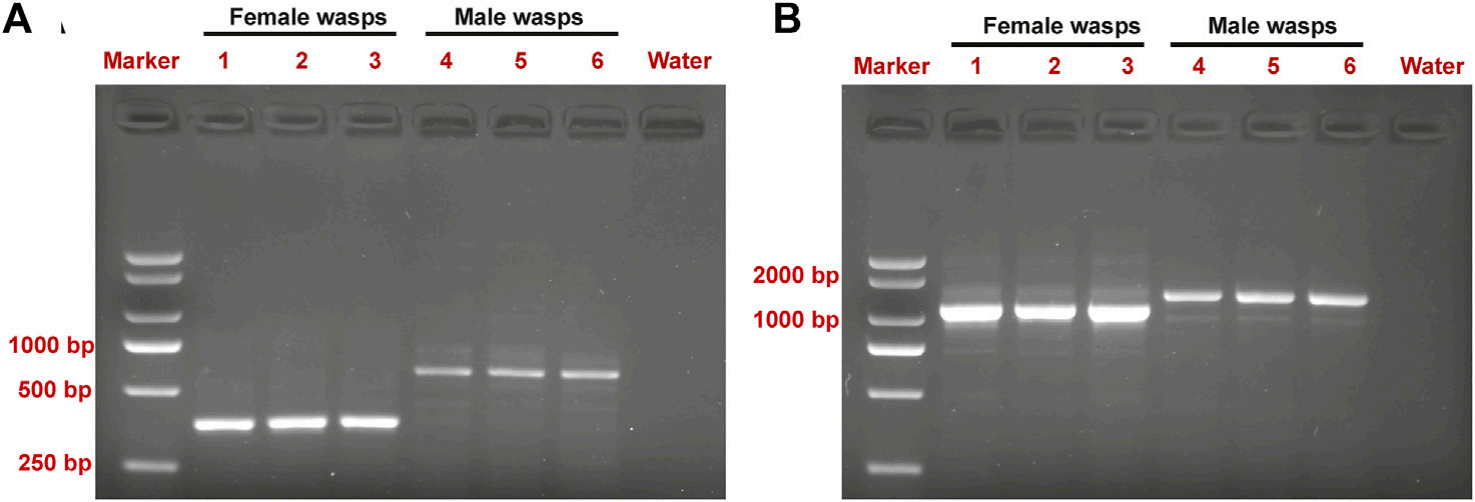
Detection of sex-specific *tra* **(A)** and *dsx* **(B)** splices from pooled cDNA samples of male and female wasps. Lane M is the DL2000 DNA marker. Lanes 1–3 in **(A)** are female-specific *tra* splices. Lanes 4–6 in **(A)** are male-specific *tra* splices. Lanes 1–3 in **(B)** are female-specific *dsx* splices. Lanes 4–6 in **(B)** are male-specific *dsx* splices.

Expression of the sex-specific *tra* and *dsx* variants was determined using the cDNA samples obtained from male and female wasps (Figure 3). Both the female-specific variant (ID: OQ847082) and male-specific variant (ID: OQ847083) of *tra* were registered on the NCBI database. The female-specific *tra* variant was composed of nine exons. The male-specific *tra* variant encoded a truncated protein consisting of 172 aa and an Arg/Ser domain. The Pro-rich and CAM domains were absent in the male-specific *tra* variant. Hence, these domains are only implicated in female-specific signals.

**FIGURE 4:**
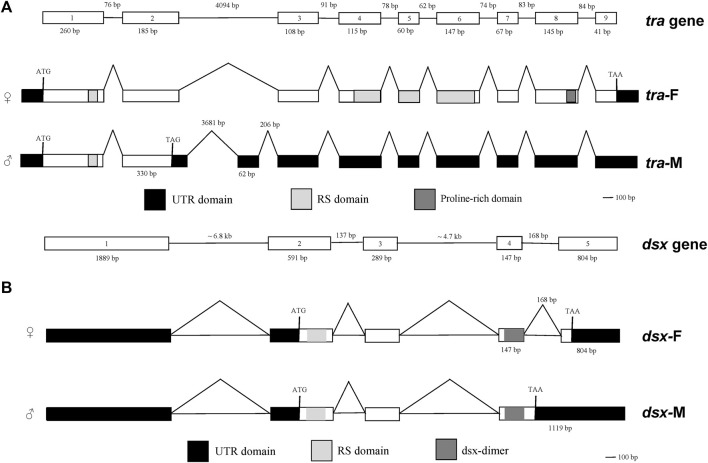
Genomic structures of sex-specific *tra* **(A)** and *dsx* splices **(B)**. Boxes represent exons. White boxes represent protein-coding exons. Black boxes represent non-translated exons. Light gray boxes indicate the arginine/serine (RS)-rich domain. Dark gray boxes in **(A)** indicate the proline (Pro)-rich domain. Dark gray boxes in **(B)** indicate the *dsx* dimer.

Both the female-specific variant (ID: OQ847080) and male-specific variant (ID: OQ847081) of *dsx* were registered on the NCBI database. *Dsx* included five exons. Female-specific exclusion was observed at the fifth exon, while male-specific exclusions occurred at the fifth exon and the fourth intron (Figure 4).

**FIGURE 5:**
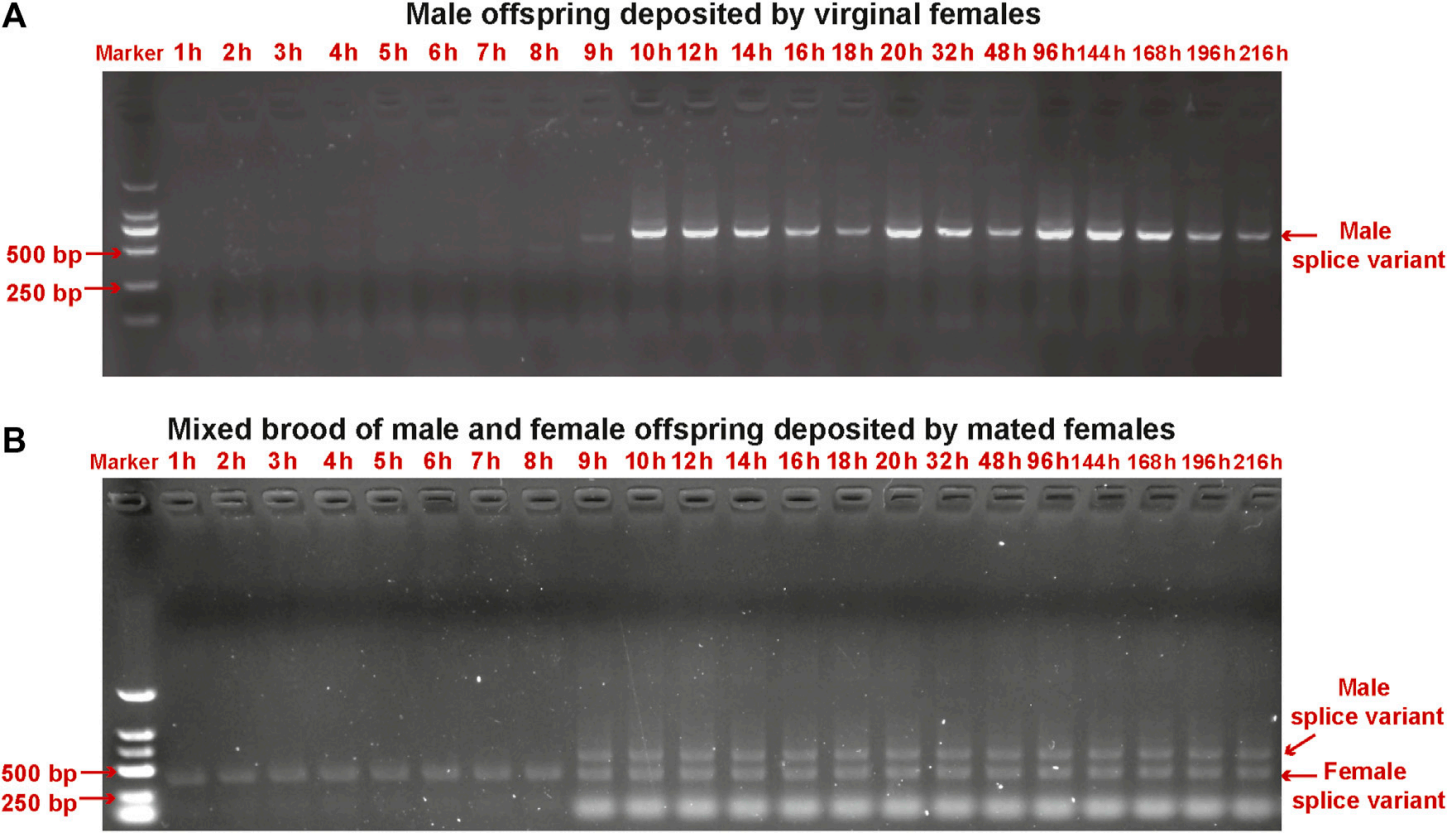
Detection of sex-specific *tra* variants in pooled samples of male offspring deposited by unmated female wasps **(A)** and mixed male and female offspring deposited by mated female wasps **(B)** collected from early embryos (at 1 h, 2 h, 3 h, 4 h, 5 h, 6 h, 7 h, 8 h, 9 h, and 10 h), late embryos (at 12 h, 14 h, 16 h, 18 h, and 20 h), larvae (at 32 h and 48 h), pre-pupae (at 96 h), and pupae (at 144 h, 168 h, 192 h, and 216 h) of *T. dendrolimi.* Lane M is the DL2000 DNA marker.

**FIGURE 6:**
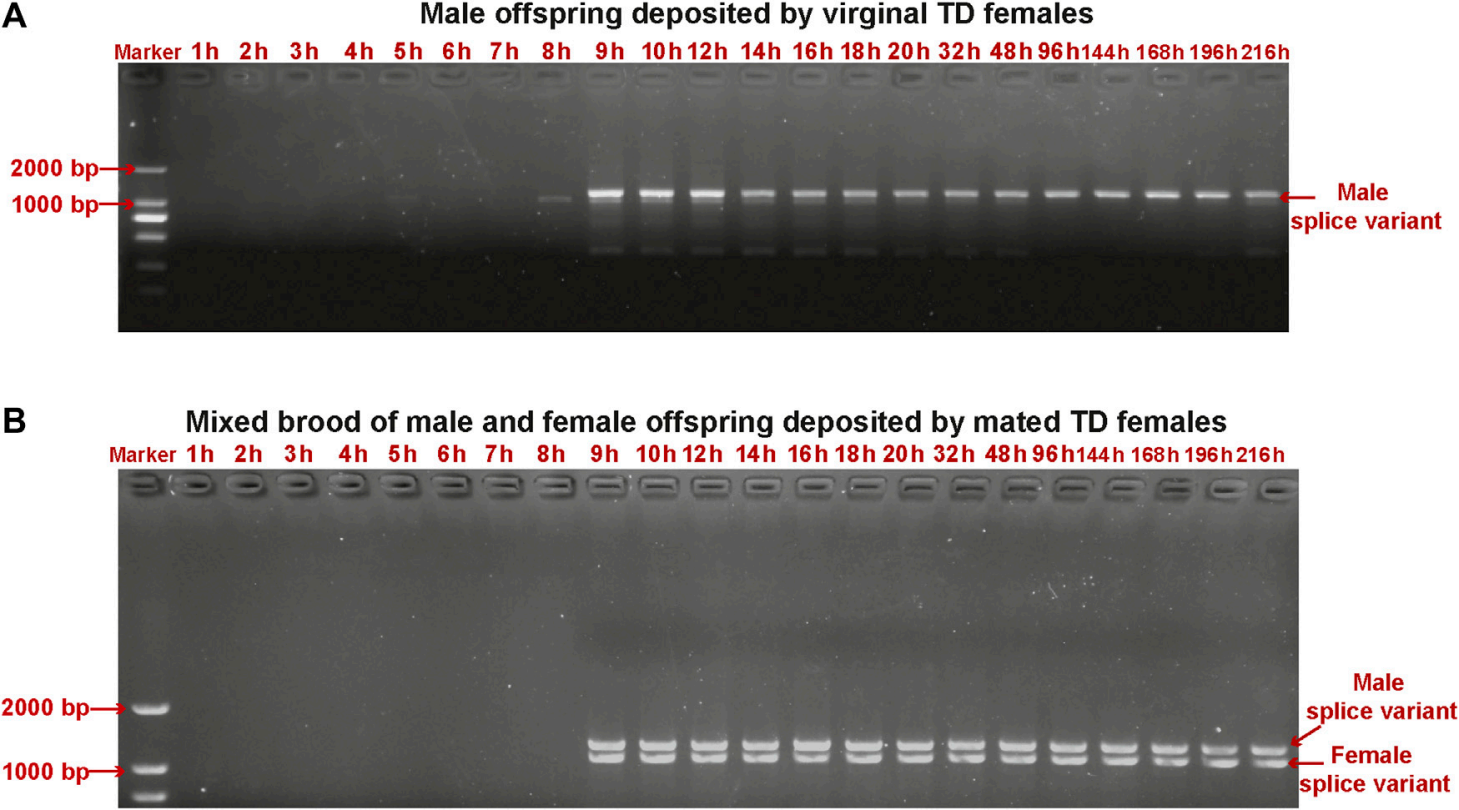
Detection of sex-specific *dsx* variants in pooled samples of male offspring deposited by unmated female wasps **(A)** and mixed male and female offspring deposited by mated female wasps **(B)** collected from early embryos (at 1 h, 2 h, 3 h, 4 h, 5 h, 6 h, 7 h, 8 h, 9 h, and 10 h), late embryos (at 12 h, 14 h, 16 h, 18 h, and 20 h), larvae (at 32 h and 48 h), pre-pupae (at 96 h), and pupae (at 144 h, 168 h, 192 h, and 216 h) of *T. dendrolimi.* Lane M is the DL2000 DNA marker.

We detected sex-specific *tra* and *dsx* variants in pooled male and mixed male–female progeny samples. Male-specific *tra* variant was observed in the male offspring after 9 h. Female-specific *tra* variant was found in the mixed male–female progeny samples at all developmental stages (Figure 5). Both female-specific and male-specific *dsx* variants were detected in the offspring after 9 h (Figure 6).

## Discussion

The present study identified the genes *tra*, *tra2*, and *dsx* which determine the sex of the natural enemy wasp *T. dendrolimi*. Sex-specific *tra* and *dsx* variants were detected in the male and female wasps. To the best of our knowledge, the present study may be the first to conduct a systematic analysis of the genes that determine sex in *Trichogramma* wasps.

In hymenopterans, the *tra*/*fem* orthologs have widely diverged, whereas those of *tra2* and *dsx* are relatively more conserved. These factors might partially explain the observed variation among different insect species in terms of their sex determination systems (Beye et al., 2003). For example, the sex determination of CSD mechanisms has been found in at least 60 species of Hymenoptera (van Wilgenburg et al., 2006). Sex-specific *tra*/*fem* splicing is governed by CSD proteins in hymenopterans (Hasselmann et al., 2008). In contrast, the CSD mechanism is virtually absent in parasitoid wasps such as *Nasonia* spp. In the members of this genus, the instructor *wom* directs *tra* splicing and is activated only in the early embryo after fertilization (Zou et al., 2020). Divergence of the *tra*/*fem* orthologs may account for the observed differences in the instructor signals among various MEGISD and CSD mechanisms.

Similar to the previously reported *Nasonia* wasps (Zou et al., 2020), the *Trichogramma* wasps presented with a female-specific *tra* variant in the early female embryo (1–20 h). The male-specific variant began to express its *tra* variant at 9–10 h as fertilization was somewhat delayed. Unlike the *Nasonia* wasps (Zou et al., 2020), the *Trichogramma* wasps presented no maternally provided female-specific *tra* variant at the early male embryo stage. Thus, the female-specific *tra* variant expressed in the female embryo at 1–9 h may not have originated from the maternal female wasp. In the *Asobara tabida* wasp, no female-specific variant was detected in the early fertilized or unfertilized eggs (Geuverink et al., 2017a). A non-sex-specific *tra* variant was detected in early *A. tabida* embryos, and it contained a putative duplicate CAM domain. However, the role of this non-sex-specific *tra* variant in *A. tabida* remains to be investigated. According to the MEGISD model, sex determination is initiated by a paternal instructor *wom*. In *Nasonia*, *wom* may only be expressed in the early embryo within several hours (Zou et al., 2020). In *Trichogramma*, the male-specific *tra* and *dsx* variants were detected and began to express, respectively, in the male embryo after 9 h. Therefore, the sex-determination mechanisms may be similar for both *Nasonia* and *Trichogramma* wasps and lack the effects of the maternally provided female-specific *tra* variant observed in *A. tabida* wasps. Nevertheless, it remains to be determined whether early sex-specific *tra* splicing is initially governed by a paternal factor such as *wom*.

To the best of our knowledge, this study may be the first case for analyzing alternative splicing of the sex determination genes *tra*, *tra2*, and *dsx* in *Trichogramma* wasps. The dynamics of sex-specific *tra* and *dsx* variant expression indicated that these sex-specific splicing events may be initially governed by an early paternal instructor such as *wom* found in *Nasonia* wasps. However, primers for the sex-specific *tra* and *dsx* variants could efficiently determine the sex of immature *Trichogramma*. The results of this work provide an important reference for investigating the sex determination mechanisms of *Trichogramma* wasps and help control the sex ratio of these natural enemy insects as they are being reared on a large scale for use in biological insect pest control programs.

## Data Availability

The original contributions presented in the study are included in the article/Supplementary Material; further inquiries can be directed to the corresponding authors.
