# Supplementary figures and images for: The identification and expression pattern of the sex determination genes and their sex-specific variants in the egg parasitoid Trichogramma dendrolimi Matsumura (Hymenoptera: Trichogrammatidae)
Source: Front Physiol. 2023 Aug 25;14:1243753. doi: 10.3389/fphys.2023.1243753 (PMC10485257; doi:10.3389/fphys.2023.1243753)

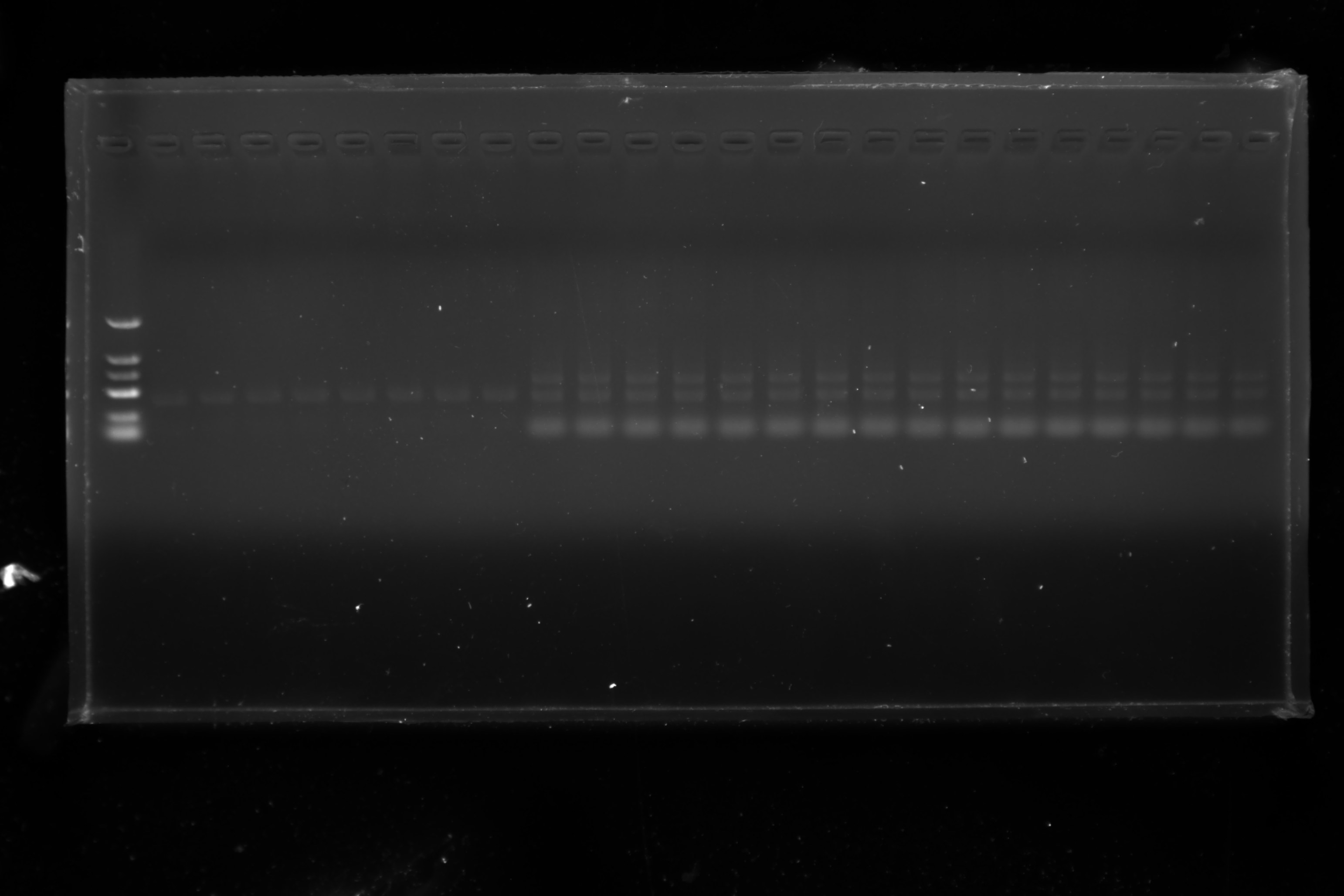

Supplement: Supplementary file 1 [file Image6.TIF]

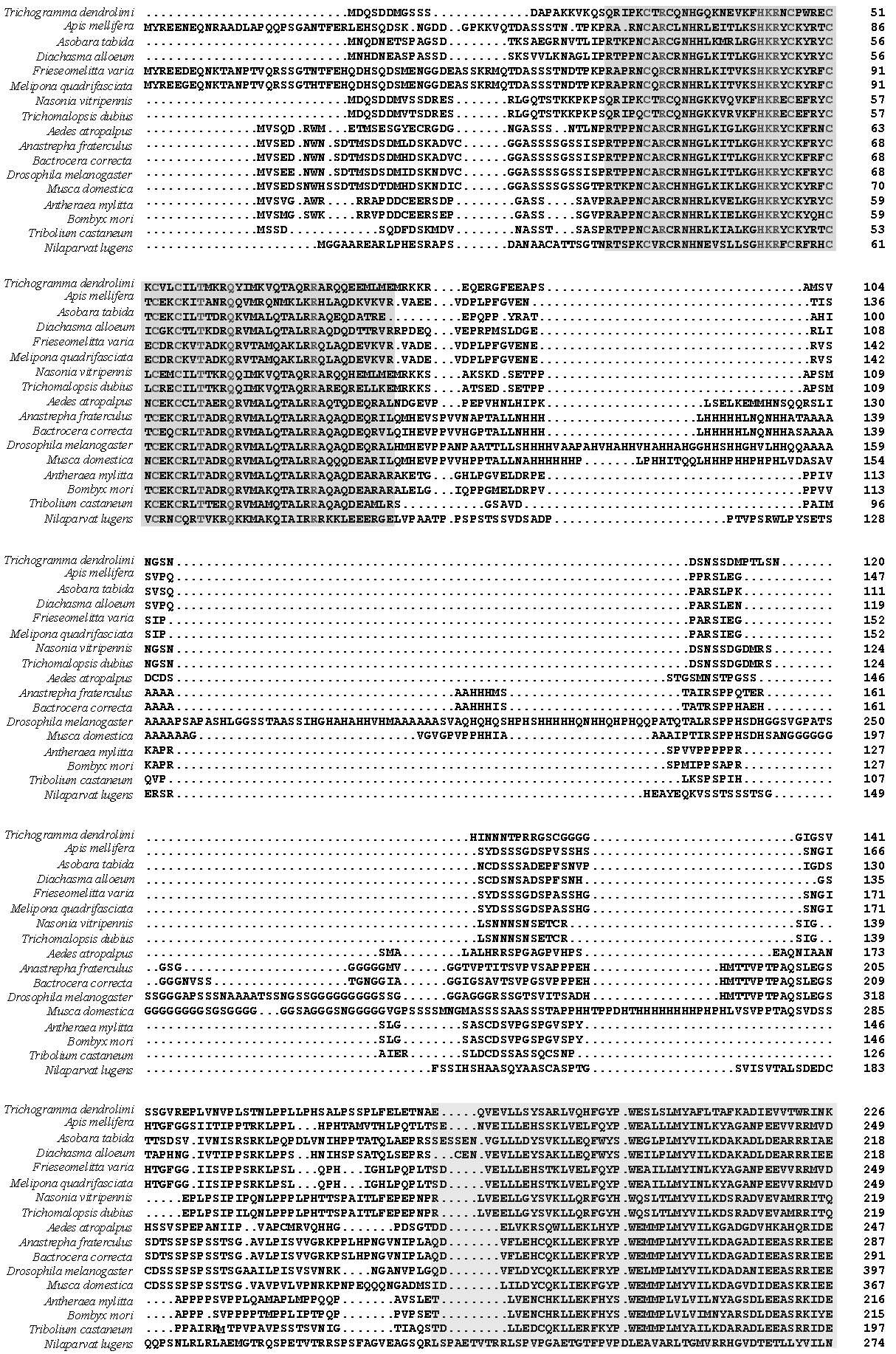

Supplement: Supplementary file 2 [file Image3.TIF]

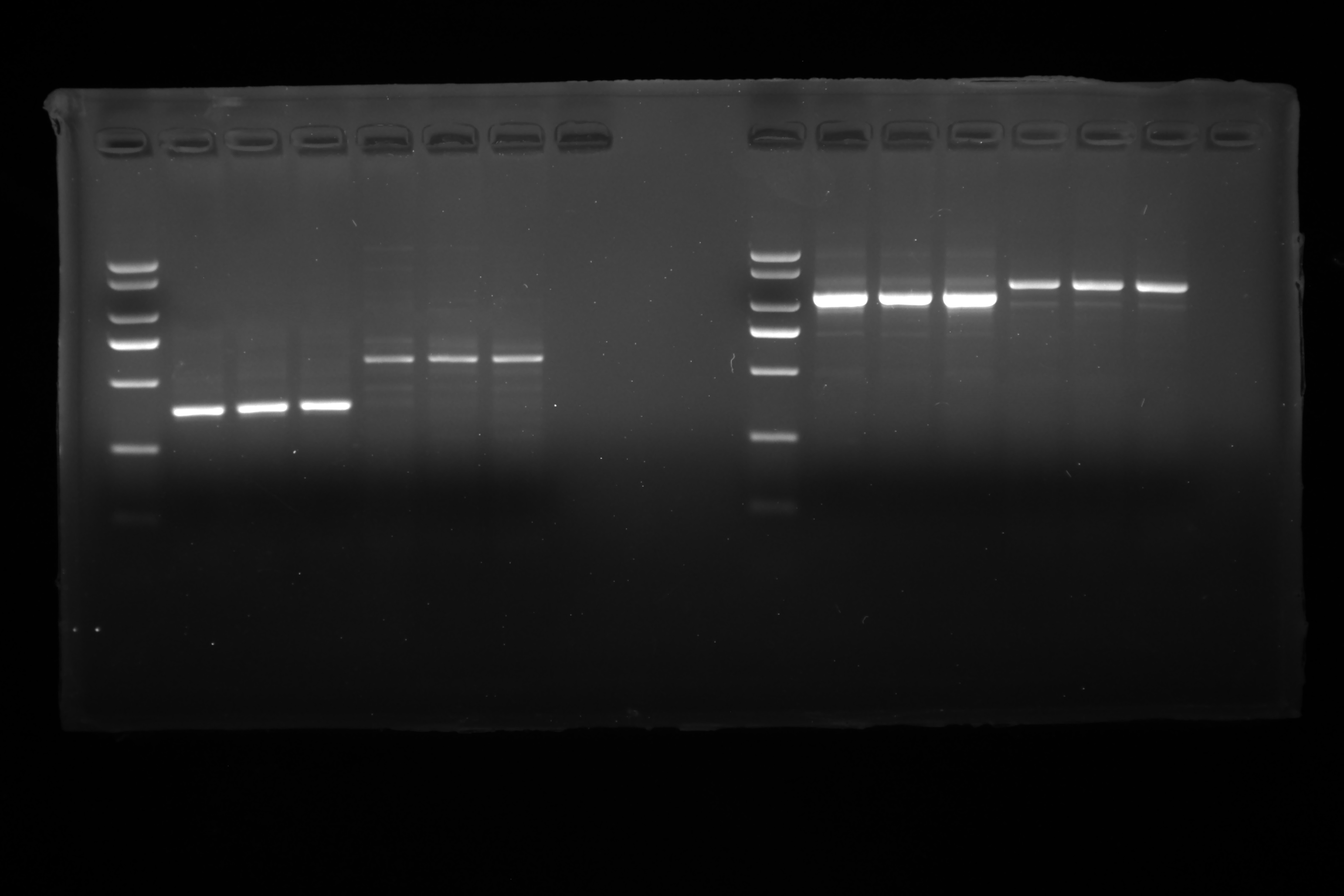

Supplement: Supplementary file 3 [file Image4.TIF]

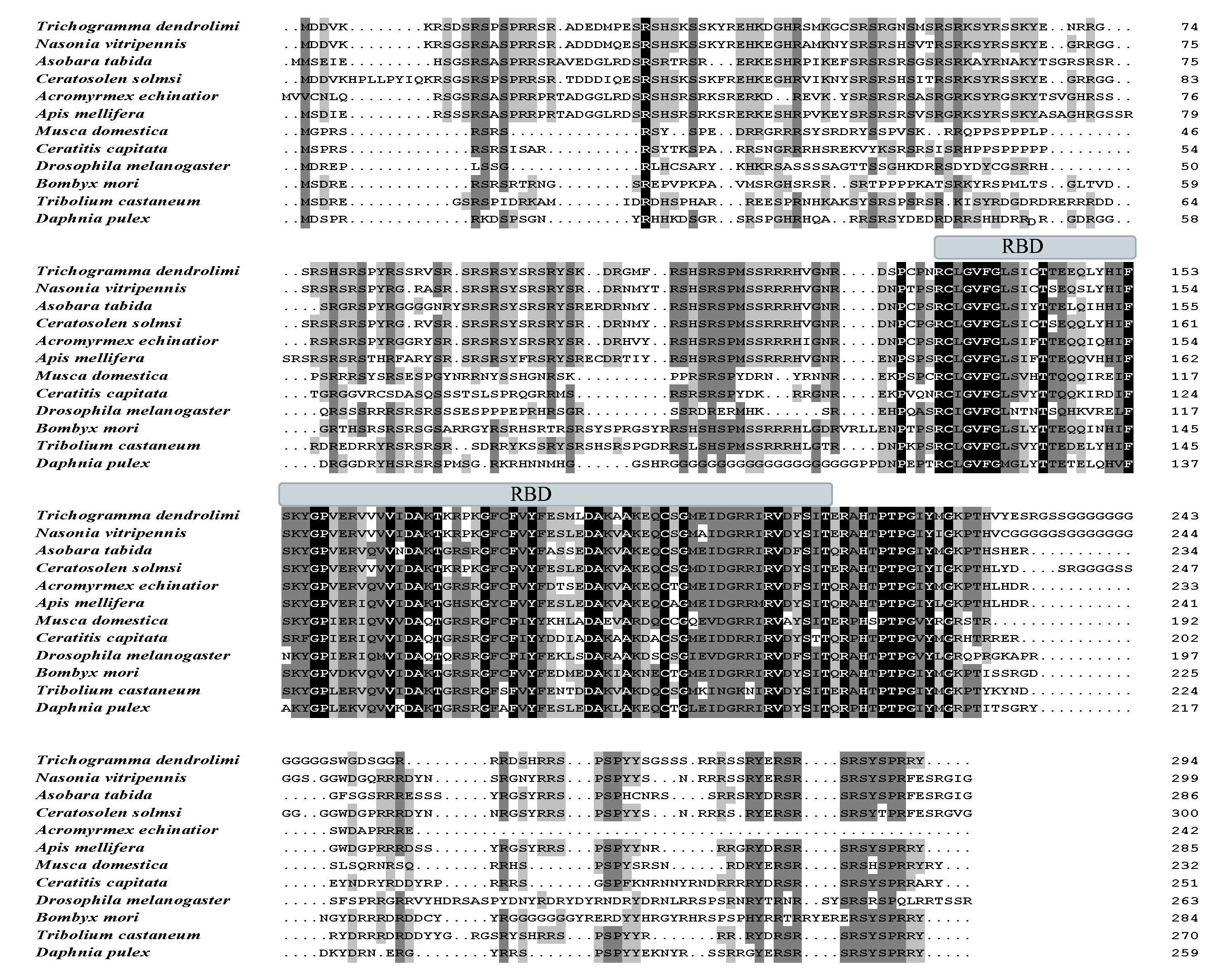

Supplement: Supplementary file 4 [file Image2.TIF]

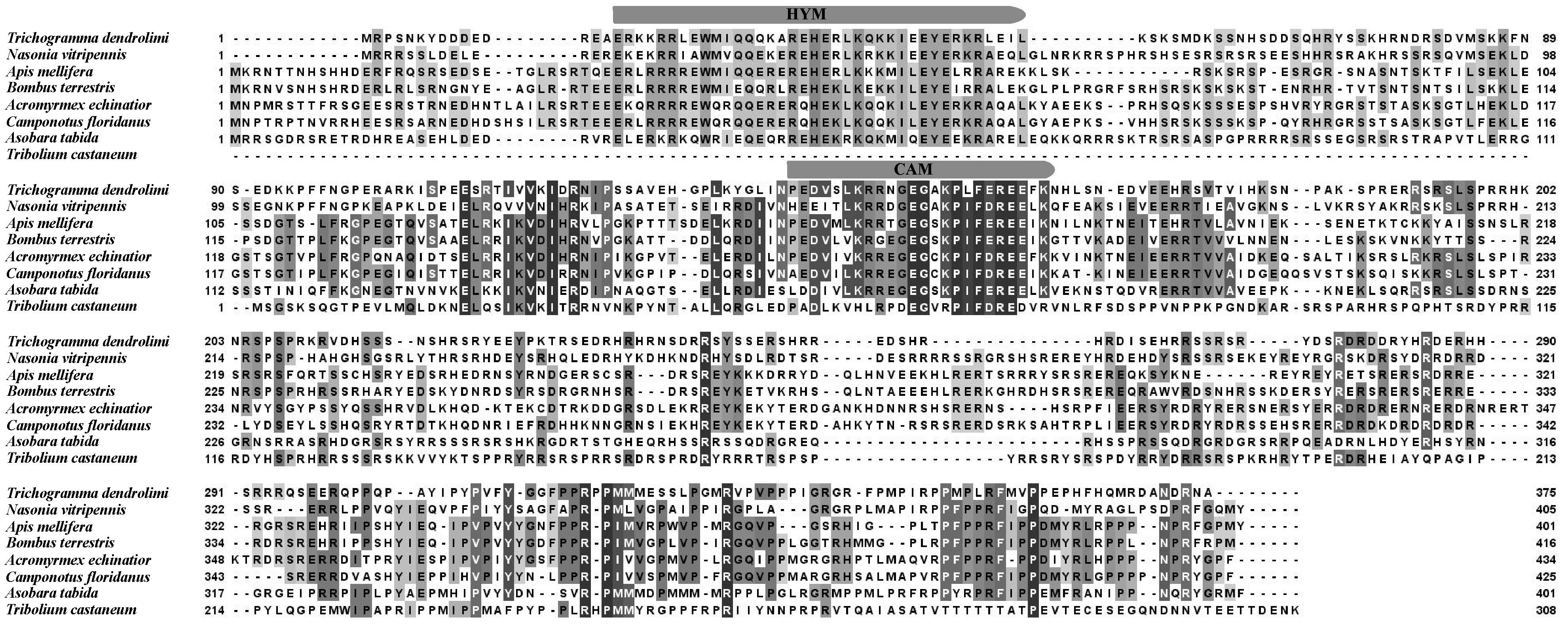

Supplement: Supplementary file 5 [file Image1.TIF]

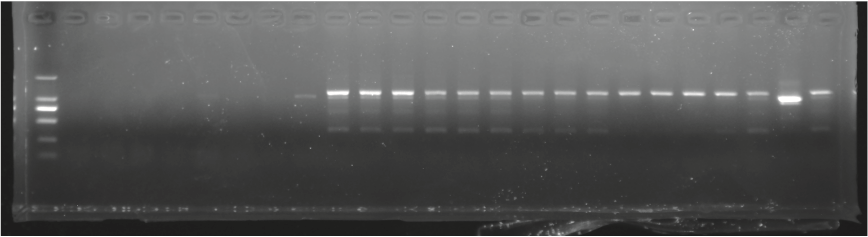

Supplement: Supplementary file 6 [file Image7.TIF]

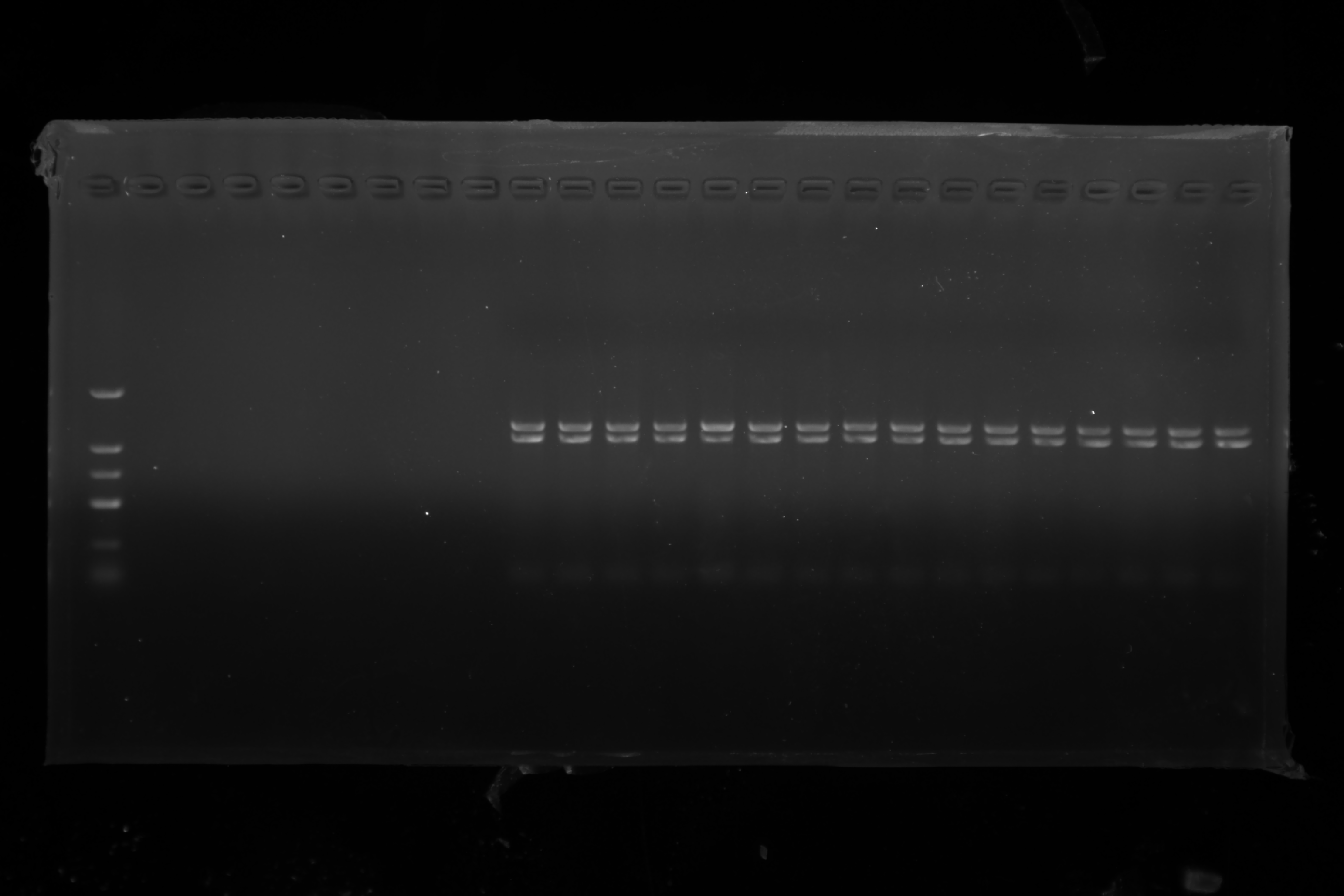

Supplement: Supplementary file 7 [file Image8.TIF]

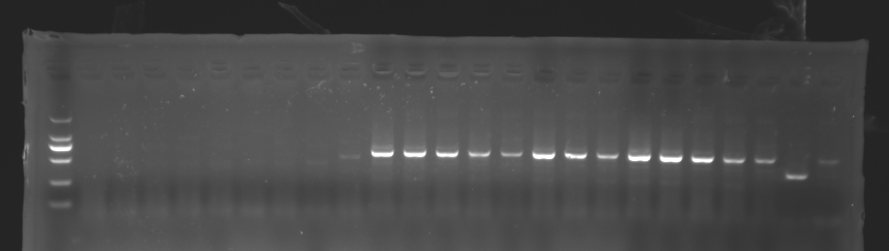

Supplement: Supplementary file 8 [file Image5.TIF]
